# Quality comparison of retorted Samgyetang made from white semi-broilers, commercial broilers, Korean native chickens, and old laying hens

**DOI:** 10.5713/ajas.19.0203

**Published:** 2019-08-03

**Authors:** Hae Seong Jeong, Dicky Tri Utama, Juntae Kim, Farouq Heidar Barido, Sung Ki Lee

**Affiliations:** 1 Department of Applied Animal Science, College of Animal Life Sciences, Kangwon National University, Chuncheon 24341, Korea; 2 Faculty of Animal Science, Universitas Brawijaya, Malang 65145, Indonesia

**Keywords:** Chicken Soup, Consumer Acceptance, Hanhyup-3-ho, Ross, Hyline White, Product Diversification

## Abstract

**Objective:**

The aim of this study was to compare the quality characteristics of retorted Samgyetang (Korean ginseng chicken soup) made from white semi-broilers (WSB; Ross× Hyline white, 3 weeks old), commercial broilers (CB; Ross, 4 weeks old), Korean native chickens (KNC; Hanhyup-3-ho, 12 weeks old) and old laying hens (OLH; Hyline white, 72 weeks old) and to explore the possibility of using the carcasses of KNCs and OLHs as raw material for product diversification.

**Methods:**

Raw and cooked meat quality, fatty acid composition and consumer acceptance were analyzed.

**Results:**

Among the chicken breeds, OLH and KNC showed a higher shear force value than WSB and CB due to high insoluble collagen contents. However, the meat of KNC was more tender than that of OLH. The meat of OLH was characterized by the lowest moisture content and highest crude fat content. The meat of KNC was characterized by a higher proportion of saturated fatty acids, α-linolenic acid and arachidonic acid than that of OLH. The meat of OLH showed the highest content of unsaturated fatty acid, particularly linoleic acid, in its thigh meat. Electronic nose readings revealed that the meat aroma pattern was clearly different across breeds. OLH had the lowest overall acceptance score, while no differences were found in flavor, texture, juiciness and appearance among WSB, CB, and KNC.

**Conclusion:**

KNC shows potential as raw material for Samgyetang, while additional preprocessing methods, such as tenderization and fat removal, are required for the utilization of OLH as raw material for retorted Samgyetang.

## INTRODUCTION

Samgyetang (ginseng chicken soup) is a traditional Korean chicken soup that is cooked using a whole chicken carcass stuffed with glutinous rice, dried jujube, ginseng, and garlic. In the past, Samgyetang was made for health recovery during the hot summer season. Currently, rapid economic changes have lead consumers to consume ready-to-eat Samgyetang because it requires a long time to prepare at home [1]. Therefore, food industries have started producing retort-pouched Samgyetang as a home meal replacement (HMR) product for convenience. As a result, HMR-type retorted Samgyetang is favored not only by local consumers but also foreigners. The convenience of retort-pouched Samgyetang for single-person households, the Korean wave and a healthy food image have generated revenue from both domestic and international markets. Currently, Samgyetang is exported to various countries, including Japan, the USA, Taiwan, Hong Kong, China, Vietnam and Australia. The export amount (HS Code 1602.32.1010) has steadily increased from 7,905,166 USD in 2013 to 11,024,202 USD in 2017. As the number of domestic single-person households is increasing, the economic value of HMR product such as Samgyetang is expected to grow continuously [2,3].

The white semi-broiler (WSB) is a crossbred chicken produced locally from a male broiler (Ross) and a female HyLine broiler. This chicken breed has white feathers, a small size, and low-fat meat and is mainly (60% to 70% of market share of Samgyetang) used as raw material to manufacture Samgyetang [4]. The meat of WSBs is more favorable than that of other chicken breeds by domestic consumers due to its cheaper price, higher stability at high-temperature processing, and chewy texture [5]. Additionally, the WSB has a great advantage for rapidly supplying raw material in the summer when the demand for Samgyetang is sharply increasing. However, the WSB is an unofficial breed produced by private industries to meet seasonal demand. Furthermore, safety issues have been raised regarding the production process.

On the other hand, Korean native chickens (KNCs) are slow to grow compared with commercial broilers (CBs), the breeding period is long, and small breeding is performed in individual farms [6]. Indigenous chickens have a unique flavor and texture that is more suitable for domestic consumers than that of CBs [7]. Compared with CBs, the meat of KNCs has less fat and a firmer texture, and it has excellent flavor because it contains a large amount of amino acids and inosinic acid, a umami-taste enhancer [8]. According to the findings of Jayasena et al [9], the meat of KNCs for Samgyetang has higher protein and lower fat contents and was less enjoyable overall than that of CBs. Old laying hens (OLHs), an egg-type chicken breed, are slaughtered when their performance declines. The meat of OHLs is difficult to sell directly to the consumer because of its low quality [10]. In recent years, studies have been undertaken to process the meat from OLH into meat products to promote consumption and provide added value [11–13]. Therefore, the aim of this study was to better understand and provide scientific information regarding the utilization of different chicken breeds to manufacture retorted Samgyetang, which is expected to be used as basic information for the diversification of Samgyetang products.

## MATERIALS AND METHODS

### Raw material

In total, 100 carcasses from four different chicken breeds—the WSB (Ross×Hyline white, 3 weeks old), CB (Ross, 4 weeks old), KNC (Hanhyup-3-ho, 12 weeks old), and OLH (Hyline white, 72 weeks old)—were purchased at 24-h post slaughter from a local slaughterhouse with average weights of 550±20 g, 750±50 g, 1,800±100 g, and 1,300±100 g, respectively. The carcass was cut in half for pouch adjustment and analysis purposes. Ten half-carcasses from each treatment group were used for raw meat quality analysis, while the others were used to make Samgyetang.

### Retorted Samgyetang manufacture

The broth for Samgyetang was prepared using 180 g of A*stragalus membranaceus* root, 300 g of Mulberry branch, 276 g of *Kalopanax septemlobus* branch, 60 g of licorice, 324 g of Siberian ginseng, and 210 g of salt per 36 L of water, followed by boiling for approximately 1 hour. The solids in the broth were removed, and the broth was filtered using a stainless mesh filter (8 mm). The final salt concentration was adjusted to 0.6% using a salinity meter (PAL-03S, Atago Co., Ltd., Tokyo, Japan) and by adding drinking water.

To prepare the stuffing, glutinous rice was soaked for 1 h and rinsed prior to use. Garlic, ginseng, and dried jujube were rinsed with cold water. Approximately 35 g of glutinous rice, 8 g of garlic, 5 g of jujube and 7 g of ginseng were placed in rice paper, which was soaked with warm water, and wrapped. The wrapped rice, breast and thigh meat were then stuffed into a retort pouch (length 19 cm [length]×25 cm [height], polyethylene terephthalate = 16 μm, aluminum = 9 μm, nylon = 15 μm, polypropylene =100 μm) gifted from Sunbong Food (Incheon, Korea) and were filled with broth until the weight reached 1,000±0.05 g. The pouches were then sealed (WB-1150VP; Woobin Tech Co., Ltd., Incheon, Korea). Two extra pouches were used to measure the F0 value during the retorting process. The retort process was performed using a steam-type retort sterilization chamber (Steri-ace, Kyungshan Co., Ltd., Gyeongsan, Korea). The heating process was started at 65°C, and the temperature was raised to 121°C for the first sterilization (pressure = 1.5 kgf/cm^2^; holding time = 3 min). Next, the temperature was raised again to 122°C for 10 s for the second sterilization (pressure = 1.7 kgf/cm^2^; holding time = 95 min). The cooling process was held at 1.7 kgf/cm^2^ for 20 min. Retort-pouched Samgyetang was stored in a refrigerator (5°C±0.5°C) prior to analysis (within 3 days). For analysis, 10 pouches of retorted Samgyetang from each treatment group were used for meat quality analysis, while the other 28 pouches from each treatment group were used for sensory evaluation.

### Proximate composition

The proximate composition of breast and thigh meat was measured according to AOAC methods [14]. The moisture content was determined by weight loss after 24 h of drying at 105°C. The crude protein content was measured using Kjeltec system (2200 Kjeltec Auto Distillation Unit, Foss, Hillerød, Denmark). The crude fat content was measured by soxhlet extraction method using diethyl ether. The crude ash content was determined after burning in a muffle furnace (LEF-115S, Daihan labtech Co., Ltd., Namyangju, Korea) of 550°C.

### Total and insoluble collagen contents

The total and insoluble collagen contents of the raw meat were measured according to Jayasena et al [9]. The sample was hydrolyzed using the method of Palka and Daun [15]. The hydrolysate was then neutralized with 10 M or 1 M NaOH and H_2_SO_4_ and filled with distilled water to a 50-fold dilution. Next, 4 mL of diluted hydrolysate and 2 mL of chloramine T solution (1.41 g of chloramine T, 10 mL of distilled water, 10 mL of n-propanol, and 80 mL of citric buffer at pH 6.0) in a test tube were mixed by vortexing and then were allowed to stand at room temperature for 20 min. Thereafter, the sample was incubated in a 60°C water bath after 2 mL of 4-dimethyl-aminobenzaldehyde solution (10 g of 4-dimethylamino-benzaldehyde, 35 mL of HCIO_4_ [60%], and 65 mL of isopropanol) was added and shaken. After cooling for 5 min in tap water, the absorbance (UV-semi-1240; Shimadzu, Kyoto, Japan) was measured at 558 nm. The hydroxyproline content was calculated by comparing with the standard curve, and the collagen content was calculated by multiplying the hydroxyproline content by a factor of 7.25. The calculated collagen content was expressed as mg/g. The extraction of insoluble collagen was performed according to the method by Liu et al [16]. The experimental procedure, after extracting insoluble collagen, was similar to that for total collagen mentioned above.

### pH, water holding capacity, and cooking loss

The pH values were measured by the homogenate with a pH meter probe (Seven Easy pH; Mettler-Toledo GmbH, Schwerzenbach, Switzerland). The homogenate was prepared with 5 g of meat sample and 45 mL of distilled water using a homogenizer (PH91; SMT Co., Ltd., Tokyo, Japan).

The water-holding capacity (WHC) was determined using a centrifugal method [17]. Approximately 5 g of the sample was placed in a centrifuge tube equipped with a wire mesh of 40 and was heated in a water bath at 75°C for 30 min. Thereafter, the sample was cooled in ice water for 10 min and centrifuged (CS-6R Centrifuge; Beckman Instruments Inc., Hialeah, FL, USA) at 980 *g* for 10 min. The WHC was calculated by the ratio of the total moisture content of the sample to the remaining water content after straining.

The cooking loss was measured using the weight of the carcass before and after the retort process. The contents in the retort-pouched Samgyetang were moved into a porcelain bowl and reheated using a microwave for 20 min (MS23F301TAW 1100W, Samsung Electronics, Suwon, Korea). After reheating, the broth and other solids except the carcass were removed using a filter tray with 40 mesh and cooling for 20 min. The carcass was weighted after removing the remaining broth on the surface using a kitchen towel carefully.

### Shear force analysis

The shear force values were measured using a texture analyzer (TAXT2*i* version 6.06, Stable Micro Systems Co., Ltd, Goldaming, UK) equipped with Warner-Bratzler shear blade. The samples were cut into cubes (2 cm×1 cm×1 cm). The cube was cut through against the muscle fibers. The conditions of measurement were as follows: load cell = 5 kg, pretest speed = 5.0 mm/s, test speed = 2.0 mm/s, and posttest speed = 10.0 mm/s. The results of measurement were presented as kg force.

### Aroma pattern analysis by electronic nose

The aroma pattern was analyzed according to Utama et al [18] with slight modifications. Ground sample (2 g) was placed in a glass vial and sealed with aluminum cap equipped with PTFE/rubber septa. The gas generated in headspace was extracted by using auto sampler (HS100, Alpha MOS, Toulouse, France) after incubation at 60°C with agitation speed of 500 rpm. The 2.5 mL of gas extracted by syringe (65°C) was injected (carrier gas, flow rate, air, 150 mL/min) into the chamber of electronic nose equipped with six metal oxide sensors. The data were processed using principal component analysis (PCA, Alpha soft version 8.01 software, Alpha MOS, France).

### Fatty acid composition

The fatty acid composition of cooked breast and thigh meat was determined using a gas chromatograph (YL6500, YL Instrument, Anyang, Korea). The lipid extraction and methylation of fatty acids were carried out according to the method of Folch et al [19] and AOAC [14], respectively. The fatty acid methyl esters in hexane (1 μL) was injected into the GC port by the auto sampler (7683, Agilent Technologies, Santa Clara, CA, USA). The inlet temperature was set at 250°C with a split ratio of 100:1. Fatty acid methyl esters were separated using a WCOT-fused silica capillary column (100 m×0.25 mm i.d., 0.20 μm film thickness; Varian Inc., Palo Alto, CA, USA) with a 1.0 mL/min helium flow. The oven was programmed as follows: 150°C/1 min, 150°C to 200°C at 7°C/min, 200°C/5 min, 200°C to 250°C at 5°C/min, and 250°C/10 min. The temperature of the detector was set at 280°C. The fatty acid peaks were identified using the retention time of fatty acid standards (47015-U, Sigma-Aldrich Corp., LLC., St. Lois, MO, USA). The peak area of each identified fatty acid was used to calculate the proportion (%) against the total identified peak area.

### Sensory evaluation

The survey of consumer acceptance on retort Samgyetang made with WSB, CB, KNC, and OLH was carried out by 28 graduate students and undergraduate students of Department of Applied Animal Science, Kangwon National University. The panels were asked to give a score of from 1 to 7 (very unacceptable = 1, very acceptable = 7) for variables such as color, texture, juiciness, flavor, greasiness, overall acceptance in breast, thigh, and broth parts, respectively. The sample composed of half of breast, leg part and broth was reheated for 6 min using a microwave (MS23F301TAW 1100 W, Samsung Electronics, Suwon, Korea) prior to serve. Drinking water was provided to cleanse the palate.

### Statistical analysis

The data were analyzed using one-way analysis of variance using R-version 3.3.0 (The R-foundation for Statistical Computing, Vienna, Austria). The mean value of each group was separated using Duncan’s multiple range test at 95% significance level.

## RESULTS AND DISCUSSION

### Proximate composition

The proximate composition of the raw breast and thigh meat is shown in Table 1. The breast and thigh meat of OLH had a lower moisture content but a higher crude fat content than the others. According to Jeon et al [20], the meat of the laying hen (82 weeks old) showed a lower moisture content than CBs and was consistent with the present findings. The proximate composition of the cooked breast and thigh meat is shown in Table 2. Regardless of the meat part, the moisture content of OLH was lower than that of KNC (p<0.05), while WSB and CB had higher moisture contents than KNC. No significant difference was found between WSB and CB (p> 0.05). For breast meat, the crude protein content of OLH was higher than that of the others (p<0.05). The highest crude protein content was observed in OLH. For thigh meat, the crude protein content of OLH was higher than those of WSB and CB but similar to that of KNC. The crude fat content of KNC was the lowest among the others (p<0.05). In general, the meat proximate composition of retorted Samgyetang showed a similar tendency to that of the raw chicken meat. Therefore, the proximate composition in retorted Samgyetang is due to that of raw chicken meat.

### pH, water holding capacity, and cooking loss

Regardless of the meat part, the pH values of raw meat showed no significant differences among the chicken breeds (Table 1). Sung et al [21] reported that the meat pH of Korean native chickens decreased with increasing age. In this study, OLH (96 weeks old) and KNC (9 weeks old) showed a lower pH value than WSB (4 weeks old) and CB (5 weeks old). Therefore, it was presumed that older chicken had a lower meat pH. The cooked breast meat pH of KNC was similar to that of WSB and CB but higher than that of OLH (Table 2). The lowest pH value of thigh meat was found in KNC, and the highest was observed in CB (p<0.05). This tendency was similar to the pH value in raw meat (Table 1). The pH value is related to meat protein and has a great influence on WHC [22]. For breast meat, the WHC of OLH was the lowest among others and no differences were found between WSB, CB and KNC. A similar trend was observed in cooking loss (Table 2). OLH indicated the largest loss (shrinkage) after retort processing, followed by KNC, CB, and WSB without any significant differences. The cooking loss is closely related to the yield and juiciness of the cooked meat. In the case of retort products, there was no weight loss of the whole product, a phenomenon more closely related to quality than yield. The higher the cooking loss was, the less juicy the cooked meat was observed to be. Thus, KNC was considered as juicy as WSB and CB.

### Tenderness

For breast meat, the lowest shear force values were observed in WSB and CB. OLH showed the highest shear force value followed by KNC (Table 2). However, no significant differences were found among the thigh meat of WSB, CB, and KNC. The thigh meat of OLH showed the highest shear force value. The shear force value is affected by age and breed and can be attributed to the collagen content [23]. Therefore, the meat tenderness of Samgyetang made from OLH was the lowest among the others. Meanwhile, the breast meat of KNC shared similar tenderness with that of WSB and CB.

### Total and insoluble collagen contents

The total collagen content of raw breast meat was not significantly different among the breeds, but the thigh meat of OLH showed the highest total collagen content (Table 1). The insoluble collagen content in the breast meat of KNC and OLH was higher than that of WSB and CB. The highest insoluble content in thigh meat was observed in OLH, followed by KNC, WSB and CB. Jayasena et al [9] reported that 7-week-old Korean native chickens had higher total collagen and insoluble collagen content than 14-week-old Korean native chickens. On the other hand, Jeon et al [8] reported that the content of collagen was significantly higher in Korean native chickens than in CBs. According to Nakamura et al [23], the remaining insoluble collagen contributes more to the year after heating. Kong et al [24] reported that cooking loss, shrinkage, and collagen solubility were significantly correlated with the shear force value of the meat. Therefore, it is suggested that the higher shear force values of the breast meat of OLH and KNC were due to the high insoluble collagen content.

### Fatty acid composition

Table 3 shows the fatty acid composition of the breast and thigh meat of retorted Samgyetang made from different chicken breeds. In breast meat, KNC showed the highest proportion of saturated fatty acid (SFA), while OLH showed the lowest (p<0.05). Most of the SFA in breast meat comprised palmitic acid (C16:0) and stearic acid (C18:0). Palmitic acid in OLH was significantly lower than that in KNC (p<0.05). On the other hand, most of the constituents of unsaturated fatty acids (USFA) were oleic acid (C18:1n9) and linoleic acid (C18:2n6) regardless of the meat part. Linoleic acid is an essential fatty acid that cannot be produced by the body along with α-linolenic acid and arachidonic acid [25]. The highest linoleic acid content was observed in the breast meat of OLH (p<0.05). The proportions of α-linolenic acid and arachidonic acid in the breast meat of WSB, CB, and KNC were higher than those in OLH. In thigh meat, the ratio of SFA in WSB and CB was similar to that in KNC, while the ratio of SFA in OLH was the lowest (p<0.05). The highest proportion of polyunsaturated fatty acid (PUFA) was found in OLH, while that of WSB, CB, and KNC was not significantly different. Linoleic acid was the most abundant USFA, and its proportion was higher in the thigh meat of OLH than in the thigh meat of the others (p<0.05). The proportions of α-linolenic acid and arachidonic acid of KNC were higher than those of OLH and similar to those of WSB and CB. The proportion of α-linolenic acid was higher in the thigh meat of CB and KNC than in that of WSB and OLH. The meat of KNC contained a higher proportion of arachidonic acid than that of other chicken breeds. Jeon et al [8] reported that the CBs had a higher content of linoleic acid and α-linolenic acid and a lower content of arachidonic acid than Korean native chickens. Lee et al [26] reported that arachidonic acid and docosahexaenoic acid of Korean native chickens showed higher rates than CBs.

### Aroma pattern

**Figure 1:**
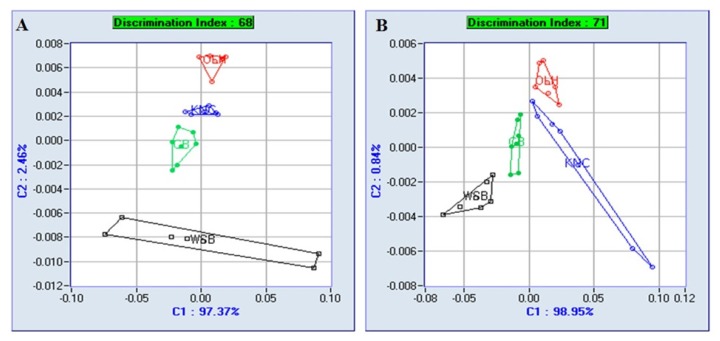
Aroma pattern analysis of breast (A) and thigh (B) meat of retorted Samgyetang made from different chicken breeds. WSB, retorted thigh meat from white-semi broilers; CB, retorted thigh meat from commercial broilers (Ross broiler); KNC, retorted thigh meat from Korean native chickens (Hanhyup-3-ho); OLH, retorted thigh meat from old layer hen (Hy-line).

The aroma pattern of breast and thigh meat in retorted Samgyetang made from WSB, CB, KNC, and OLH is shown in Figure 1. Regardless of the meat part, the aroma pattern of OLH, KNC, CB, and WSB formed separately without overlap from top (OLH) to bottom (WSB). The discrimination indices of the breast and thigh meat were 68 and 71, respectively, indicating differences between the groups. As the number decreases to negative, it indicates no difference between the aroma groups, while a positive indicates a difference. The generation of aroma compounds is complicatedly influenced by various factors. Thermal lipid degradation and the Maillard reaction are known to have a major influence on flavor, and approximately 500 volatile compounds were identified in chicken meat [27]. The reaction of cysteine and sugar can lead to characteristic meat flavor, especially for chicken and pork [9]. According to Elmore et al [28], cooked beef steaks with high level of PUFA generated higher levels of lipid oxidation products such as n-alkanals, 2-alkenals, 1-alkanols, and alkylfurans up to 4-fold.

### Consumer acceptance

The consumer acceptance scores of the breast and thigh meat of retorted Samgyetang made from WSB, CB, KNC, and OLH are shown in Table 4. In breast meat, the scores of all traits except color were higher in WSB, CB, and KNC than in OLH (p<0.05), and no significant difference was found among WSB, CB, and KNC. In thigh meat, the scores of color, texture, juiciness, and flavor in WSB, CB, and KNC were similar but were higher than those in OLH. There was no significance difference in the score of flavor between the thigh meat of KNC and OLH. Accordingly, the scores of WSB and CB were the highest and those of OLH were the lowest regarding the overall acceptance (p<0.05). In the case of broth, the scores for WSB, CB, and KNC were entirely higher than those for OLH in all traits except flavor (p<0.05). Particularly, the score for the greasy trait in OLH was the lowest, indicating that consumers did not like the greasy broth in that sample as the meat of OLH contained a higher fat content than the others.

## CONCLUSION

The retorted Samgyetang made from WSB and CB were similar in physicochemical characteristics and consumer acceptance scores each other and showed better quality than retorted Samgyetang made from KNC and OLH. So, CB can be used for raw material for retorted Samgyetang on behalf of WSB. The retorted Samgyetang made from OLH showed lower acceptance than that made from KNC due to the higher fat content in the meat, a greasy broth, and tough meat. Therefore, additional preprocessing methods, such as tenderization and fat removal, are required for the utilization of OLH as raw material for retorted Samgyetang. The retorted Samgyetang made from KNC showed the level between CB and OLH regarding the moisture content and tenderness level, but there were no differences in the consumer acceptance score between KNC and CB. Additionally, the content of α-linolenic acid and arachidonic acid, which are essential fatty acids, was high in KNC. Therefore, KNC (Hanhyup-3-ho) was considered as for raw material for differentiated retorted Samgyetang compared to existing retorted Samgyetang, which made from WSB, maintaining acceptance.

## Figures and Tables

**Table 1:** Meat physicochemical properties of raw breast and thigh from different chicken breeds

Parameters	Treatments1)				SEM

	WSB	CB	KNC	OLH	
Breast					
Moisture (%)	75.0^ab^	76.1^a^	75.9^a^	73.5^b^	0.40
Crude protein (%)	23.2^ab^	21.9^b^	22.4^ab^	24.5^a^	0.26
Crude fat (%)	0.60^b^	0.70^b^	0.60^b^	1.10^a^	0.07
Ash (%)	1.20	1.30	1.20	1.10	0.03
pH	6.26	6.23	6.10	5.90	0.06
WHC (%)	75.4^a^	71.4^ab^	72.5^ab^	65.5^b^	1.50
Total collagen (mg/g)	1.73	1.55	1.59	1.58	0.04
Insoluble collagen (mg/g)	0.50^b^	0.53^b^	0.74^a^	0.84^a^	0.05
Thigh					
Moisture (%)	74.9^a^	75.3^a^	75.2^a^	72.4^b^	0.45
Crude protein (%)	19.5	18.6	19.3	20.0	0.24
Crude fat (%)	4.90^ab^	5.10ab	4.50^b^	6.60^a^	0.34
Ash (%)	1.00	1.10	1.10	1.00	0.02
pH	6.97	6.86	6.58	6.75	0.07
WHC (%)	69.7^a^	67.2^a^	68.9^a^	54.0^b^	2.39
Total collagen (mg/g)	2.09^b^	1.74^b^	2.10^b^	3.36^a^	0.22
Insoluble collagen (mg/g)	0.74^b^	0.70^b^	1.03^ab^	1.26^a^	0.08

SEM, standard error of the mean; WHC, water holding capacity.

1) WSB, white-semi broilers; CB, commercial broilers (ross broiler); KNC, Korean native chickens (Hanhyup-3-ho); OLH, old laying hen (Hy-line).

a–c Means within each row are significantly different (p<0.05).

**Table 2:** Meat physicochemical properties of retorted Samgyetang made from different chicken breeds

Parameters	Treatments1)				SEM

	WSB	CB	KNC	OLH	
Breast					
Moisture (%)	66.7^a^	67.2^a^	65.6^b^	61.4^c^	0.73
Crude protein (%)	29.7^b^	29.6^b^	31.0^b^	33.7^a^	0.52
Crude fat (%)	3.20^b^	2.80^b^	3.00^b^	4.70^a^	0.27
Ash (%)	0.70	0.80	0.70	0.70	0.03
pH	6.37^a^	6.37^a^	6.21^a^	5.99^b^	0.06
Shear force (kg)	1.61^c^	1.45^c^	2.22^b^	2.49^a^	0.10
Thigh					
Moisture (%)	65.6^a^	65.8^a^	63.4^b^	61.0^c^	0.64
Crude protein (%)	25.2^b^	24.7^b^	28.3^ab^	28.9^a^	0.64
Crude fat (%)	8.80^ab^	9.20^a^	7.60^b^	9.90^a^	0.32
Ash (%)	0.90	0.70	0.80	0.60	0.05
pH	6.88^ab^	6.92^a^	6.74^b^	6.88^ab^	0.03
Shear force (kg)	0.88^b^	0.84^b^	0.95^b^	2.87^a^	0.18
Whole carcass					
Cooking loss (%)	34.3^b^	35.0^b^	36.8^ab^	39.4^a^	0.61

SEM, standard error of the mean.

1) WSB, white-semi broilers; CB, commercial broilers (ross broiler); KNC, Korean native chickens (Hanhyup-3-ho); OLH, old laying hen (Hy-line).

a–c Means within each row are significantly different (p<0.05).

**Table 3:** Fatty acid composition (%) of retorted Samgyetang made from different chicken breeds

Fatty acid composition (%)	Treatments1)				SEM

	WSB	CB	KNC	OLH	
Breast					
C14:0 (Myristic acid)	0.93	0.95	1.17	1.05	0.06
C16:0 (Palmitic acid)	24.9^ab^	24.5^b^	26.1^a^	24.7^b^	0.26
C16:1 (Palmitoleic acid)	4.53	5.03	4.75	3.94	0.22
C18:0 (Stearic acid)	8.61	9.02	8.54	7.73	0.21
C18:1n9 (Oleic acid)	40.5	40.2	39.7	41.5	0.54
C18:2n6 (Linoleic acid)	18.3	17.4	16.9	19.5	0.48
C18:3n6 (γ-Linolenic)	1.03^b^	1.32^a^	1.05^b^	0.76^c^	0.07
C18:3n3 (α-Linolenic acid)	0.43^a^	0.56^a^	0.44^a^	0.15^b^	0.05
C20:4n6 (Arachidonic acid)	0.49^b^	0.67^ab^	0.91^a^	0.48^b^	0.06
C22:4n6 (Docosatetraenoic acid)	0.26^b^	0.36^a^	0.39^a^	0.12^c^	0.04
Saturated fatty acid	34.4^ab^	34.4^ab^	35.8^a^	33.5^b^	0.37
Unsaturated fatty acid	65.6^ab^	65.6^ab^	64.2^b^	66.5^a^	0.37
Polyunsaturated fatty acid	20.5	20.3	19.7	21.1	0.44
Thigh					
C14:0 (Myristic acid)	0.94	1.01	1.00	1.01	0.03
C16:0 (Palmitic acid)	24.0^a^	24.2^a^	24.1^a^	22.4^b^	0.27
C16:1 (Palmitoleic acid)	5.26^ab^	5.76^a^	6.18^a^	4.27^b^	0.26
C18:0 (Stearic acid)	8.61^a^	8.16^ab^	7.31^b^	7.59^ab^	0.20
C18:1n9 (Oleic acid)	42.2	42.1	42.4	42.0	0.45
C18:2n6 (Linoleic acid)	17.0^b^	16.8^b^	17.1^b^	21.5^a^	0.68
C18:3n6 (γ-Linolenic)	1.31^a^	1.28^b^	1.12^b^	0.79^c^	0.07
C18:3n3 (α-Linolenic acid)	0.21^ab^	0.24^a^	0.23^a^	0.13^b^	0.02
C20:4n6 (Arachidonic acid)	0.34^ab^	0.28^ab^	0.42^a^	0.27^b^	0.02
C22:4n6 (Docosatetraenoic acid)	0.15^a^	0.14^a^	0.15^a^	0.04^b^	0.02
Saturated fatty acid	33.5^a^	33.4^a^	32.4^ab^	31.0^b^	0.37
Unsaturated fatty acid	66.5^b^	66.6^b^	67.6^ab^	69.0^a^	0.37
Polyunsaturated fatty acid	19.0^b^	18.7^b^	19.0^b^	22.7^a^	0.61

SEM, standard error of the mean.

1) WSB, white-semi broilers; CB, commercial broilers (ross broiler); KNC, Korean native chickens (Hanhyup-3-ho); OLH, old laying hen (Hy-line).

a–c Means within each row are significantly different (p<0.05).

**Table 4:** Consumer acceptance score for retorted Samgyetang made from different chicken breeds

Traits	Treatments1)				SEM

	WSB	CB	KNC	OLH	
Breast					
Color	5.4^a^	5.4^a^	5.0^ab^	4.3^b^	0.15
Texture	5.1^a^	5.1^a^	4.2^a^	3.1^b^	0.20
Juiciness	4.7^a^	4.8^a^	4.2^a^	3.2^b^	0.19
Flavor	5.1^a^	5.2^a^	4.7^a^	3.4^b^	0.18
Overall acceptance	5.2^a^	5.4^a^	4.6^a^	3.5^b^	0.17
Thigh					
Color	5.2^a^	5.5^a^	4.7^a^	3.6^b^	0.17
Texture	5.7^a^	5.5^a^	4.9^a^	3.9^b^	0.17
Juiciness	5.8^a^	5.4^a^	5.1^a^	3.9^b^	0.18
Flavor	5.6^a^	5.1^a^	4.8^ab^	3.9^b^	0.18
Overall acceptance	5.8^a^	5.3^ab^	4.8^b^	4.0^c^	0.16
Broth					
Color	5.2^a^	5.2^a^	4.7^a^	3.3^b^	0.18
Flavor	5.0^a^	4.9^a^	4.2^ab^	3.4^b^	0.18
Greasiness	4.3^a^	4.6^a^	3.8^a^	2.6^b^	0.21
Overall acceptance	5.0^a^	5.2^a^	4.4^a^	3.2^b^	0.18

SEM, standard error of the mean.

1) WSB, white-semi broilers; CB, commercial broilers (ross broiler); KNC, Korean native chickens (Hanhyup-3-ho); OLH, old laying hen (Hy-line).

a–c Means within each row are significantly different (p<0.05).

## References

[1] Lee JH, Lee JH, Lee KT (2014). Physicochemical and sensory characteristics of Samgyetang retorted at different F0 values during storage at room temperature. J Korean Soc Food Preserv.

[2] Korea Agro-Fisheries and Food Trade Corporation (2018). Samgyetang report for China [Internet].

[3] Kim S, Lee K, Lee Y (2018). Selection attributes of home meal replacement by food-related lifestyles of single-person households in South Korea. Food Qual Prefer.

[4] Cho JH, Um JS, Yu MS, Paik IK (2007). Effect of ME and crude protein content of diet on the performance and production cost of white semibroiler chickens. Korean J Poult Sci.

[5] Park MN, Hong EC, Kang BS, Hwangbo J, Kim HK (2011). Performance and meat quality of three-crossbreed Korean native chickens (KNC). Korean J Poult Sci.

[6] Sang BD, Kong HS, Kim HK (2006). Estimation of genetic parameters for economic traits in Korean native chickens. Asian-Australas J Anim Sci.

[7] Wattanachant S, Benjakul S, Ledward DA (2004). Composition, color, and texture of Thai indigenous and broiler chicken muscles. Poult Sci.

[8] Jeon HJ, Choe JH, Jung YK, Kruk ZA, Lim DG, Jo C (2010). Comparison of the chemical composition, textural characteristics, and sensory properties of North and South Korean native chickens and commercial broilers. Korean J Food Sci An.

[9] Jayasena DD, Jung S, Kim HJ (2013). Comparison of quality traits of meat from Korean native chickens and broilers used in two different traditional Korean cuisines. Asian-Australas J Anim Sci.

[10] Jin SK, Kim IS, Jung HJ, Kim DH, Choi YJ, Hur SJ (2007). The development of sausage including meat from spent laying hen surimi. Poult Sci.

[11] Lee SK, Kang SM, Lee IS (2010). Manufacture of spent layer chicken meat products by natural freeze-drying during winter. Korean J Food Sci Anim Resour.

[12] Kim YJ (2014). The study on the quality of sausage manufactured with different mixture ratios of spent laying hen and pork meat. Korean J Poult Sci.

[13] Baek KH, Utama DT, Lee SG, An BK, Lee SK (2016). Effects of replacing pork back fat with canola and flaxseed oils on physicochemical properties of emulsion sausages from spent layer meat. Asian-Australas J Anim Sci.

[14] Latimer GW (2012). AOAC International. Official methods of analysis of AOAC International.

[15] Palka K, Daun H (1999). Changes in texture, cooking losses, and myofibrillar structure of bovine M. *semitendinosus* during heating. Meat Sci.

[16] Liu A, Nishimura T, Takahashi K (1996). Relationship between structural properties of intramuscular connective tissue and toughness of various chicken skeletal muscles. Meat Sci.

[17] Kristensen L, Purslow PP (2001). The effect of ageing on the water-holding capacity of pork: role of cytoskeletal proteins. Meat Sci.

[18] Utama DT, Lee CW, Park YS, Jang A, Lee SK (2018). Comparison of meat quality, fatty acid composition and aroma volatiles of Chikso and Hanwoo beef. Asian-Australas J Anim Sci.

[19] Folch J, Lees M, Sloaney Stanley GH (1957). A simple method for the isolation and purification of total lipides from animal tissues. J Biol Chem.

[20] Jeon KH, Hwang YS, Kim YB (2015). Physico-chemical characteristics evaluation of spent hen and broiler. Korean J Food Nutr.

[21] Sung SK, Yang TM, Kwon YJ, Choi JD, Kim DG (2000). The quality characteristics of Korean native chicken by the age. Korean J Anim Sci.

[22] Kim BC, Park GB, Sung SK (1998). The science of muscle foods.

[23] Nakamura R, Sekoguchi S, Sato Y (1975). The contribution of intramuscular collagen to the tenderness of meat from chickens with different ages. Poult Sci.

[24] Kong F, Tang J, Lin M, Rasco B (2008). Thermal effects on chicken and salmon muscles: Tenderness, cook loss, area shrinkage, collagen solubility and microstructure. LWT-Food Sci Technol.

[25] Jandacek RJ (2017). Linoleic acid: a nutritional quandary. Healthcare.

[26] Lee KH, Kim HJ, Lee HJ, Kang MG, Jo C (2012). A study on components related to flavor and taste in commercial broiler and Korean native chicken meat. Korean J Food Preserv.

[27] Brunton NP, Cronin DA, Monahan FJ (2002). Volatile components associated with freshly cooked and oxidized off-flavours in turkey breast meat. Flavour Fragr J.

[28] Elmore JS, Mottram DS, Enser M, Wood JD (1999). Effect of the polyunsaturated fatty acid composition of beef muscle on the profile of aroma volatiles. J Agric Food Chem.

